# An Essential NRP1-Mediated Role for Tagln2 in Gastric Cancer Angiogenesis

**DOI:** 10.3389/fonc.2021.653246

**Published:** 2021-06-04

**Authors:** Hongwei Jin, Wei Zheng, Jingjing Hou, Huifang Peng, Huiqin Zhuo

**Affiliations:** ^1^Xiamen Key Laboratory of Biomarker Translational Medicine, Medical Laboratory of Xiamen Humanity Hospital Fujian Medical University, Xiamen, China; ^2^Department of Gastrointestinal Surgery, The Affiliated Zhongshan Hospital, Xiamen University, Xiamen, China; ^3^Department of Gastrointestinal Surgery, Xiamen Municipal Key Laboratory of Gastrointestinal Oncology, Xiamen, China; ^4^Department of Endocrinology, The First Affiliated Hospital and College of Clinical Medicine of Henan University of Science and Technology, Luoyang, China

**Keywords:** Tagln2, NRP1, gastric cancer, angiogenesis, tumor-derived endothelial cell

## Abstract

Knowledge about the precise biological role and underlying mechanism of Tagln2 in tumor progression is relatively limited, especially in angiogenesis focused on tumor derived endothelial cells (ECs) has rarely been reported. Here, the function, molecular mechanism and potential clinical value of Tagln2 in gastric cancer (GC) angiogenesis were investigated. GC tissue microarrays were used to assess the expression of Tagln2 in ECs. The relationships between expression and clinicopathological features were analyzed to evaluate the clinical value of Tagln2. Gain- and loss-of-function approaches were performed in ECs to investigate the functions of Tagln2 in angiogenesis. A combination of angiogenesis antibody array, RNA-Seq analyses and a series of *in vitro* experiments were performed to reveal the proangiogenic mechanism mediated by NRP1. Immunohistochemistry performed on an independent tissue chip (*n*=75) revealed significant upregulation of Tagln2 in tumor-derived ECs which were specifically immunolabeled with CD34. Additionally, high Tagln2 levels correlated significantly with the presence of lymph node as well as distant metastases. Gain- and loss-of-function approaches highlighted the function of Tagln2 in promoting EC proliferation, motility, and capillary-like tube formation and in reducing apoptosis. Tagln2 upregulation led to significantly increased mRNA and protein levels of NRP1 and subsequently activated the NRP1/VEGFR2 and downstream MAPK signaling pathways. These data indicate the importance of Tagln2 in angiogenesis, as a potential therapeutic target, and as a candidate prognostic marker in GC.

## Introduction

Gastric cancer (GC) is one of the most common malignant tumors worldwide. Its morbidity and mortality rate ranked fifth and fourth, respectively, in 2020 (1). The incidence of GC is high in China, and the number of new cases (679,100) and deaths (498,000) in 2015 ranked second only to lung cancer (LC) among all types of tumors (2). In the last decade, several antiangiogenic drugs based on the VEGF-VEGFR2 signaling axis (the most highly recognized target for current antiangiogenic therapy), including bevacizumab (a monoclonal antibody against VEGF), ramucirumab (a monoclonal antibody against VEGFR2) and apatinib (a tyrosine kinase inhibitor targeting VEGFR2), have been introduced for GC treatment. However, the survival benefit leads only to short survival benefits, and the need to investigate molecular mechanisms and potential therapeutic targets for combating angiogenesis in GC.

Tagln2 is a 22-kDa actin stress fiber-associated protein that stabilizes actin filaments, with ~70% amino acid sequence homology to two of its isoforms, Tagln1 and Tagln3. The function of Tagln2 has not yet been clarified, but recent reports have demonstrated that certain tumors, including those of colorectal cancer (3), bladder cancer (4), uterine cervical squamous cell carcinoma (5), esophageal squamous cell carcinoma (6) and gliomas (7), exhibit Tagln2 overexpression. Tagln2 upregulation is associated with lymph node and distant metastasis, advanced stage, and poor survival in colorectal carcinoma (3). In addition, suppression of Tagln2 markedly decreased cell viability by inducing apoptosis in bladder cancer cell lines (4). Moreover, *TAGLN2* is the target of certain tumor-suppressive miRNAs, such as miR-1, miR-133a (4, 6), miR-145-5p (8) and miR-133b (9). These findings suggest the roles of Tagln2 in tumor progression or metastasis. Conversely, Tagln2 is frequently downregulated in breast and prostate cancers (10). In addition, a role has been suggested for Tagln2 in impeding metastasis in metastasized Barrett’s adenocarcinoma (11). In hepatocellular carcinoma cells with PFTK1 suppression, unphosphorylated Tagln2 inhibits cell motility through its strong actin-binding ability, which possibly abolishes actin cytoskeleton dynamics (12), thereby suggesting a role as a tumor suppressor. These contradictory results reveal a complex role for Tagln2 in tumorigenesis.

Knowledge about the precise biological role and underlying mechanism of Tagln2 in tumor progression is relatively limited compared to knowledge about other Tagln isoforms. As described in previous reports, Tagln2 is essential for the formation of stable immunological synapses between cytotoxic T cells and cancer cells (13), and Tagln2-actin-lymphocyte function-associated antigen-1 (LFA-1) axis for the effects of adoptive T cell therapy (14). Tagln2 is significantly induced in hypoxic lung cancer cells, accompanied by an increase in epithelial-mesenchymal transition (EMT) and resistance to γ-radiation *via* activation of the IGF1Rβ/PI3K/AKT pathway (15). Our group found that Tagln2 expression was significantly higher in microvascular endothelial cells (ECs) from lung cancer tumor tissues than in their paired normal counterparts, which was associated with advanced clinical stage, increased tumor size, and histological neural invasion (16). These findings demonstrated the essential function of Tagln2 in tumor angiogenesis for the first time, but the precise molecular mechanism involved in this process has not yet been reported.

Here, GC tissue microarrays containing 75 tumor tissues and paired normal counterparts were used to assess the expression of Tagln2 in ECs, in which CD34 was used to specifically label ECs. The relationship between expression and clinicopathological features was analyzed to evaluate the clinical value of Tagln2. Gain- and loss-of-function experiments of Tagln2 in angiogenesis were performed in ECs, with a focus on proliferation, motility, apoptosis, and capillary-like tube formation. A combination of angiogenesis antibody array, RNA-Seq analyses and a series of cell-based functional experiments were performed to reveal the critical role of Neuropilin-1 (NRP1, the coreceptor with VEGFR2 for the VEGF_165_ isoform of VEGF-A) and downstream MAPK signaling pathways in the angiogenic activity of Tagln2.

## Materials and Methods

### Cells and Arrays

EC lines (Ealy926 and ED-25) were purchased from the Institute of Cell Biology (Shanghai, China) and cultured in DMEM-F12 medium supplemented with 10% fetal bovine serum (FBS, HyClone, Logan, UT). Primary human umbilical vein endothelial cells (HUVECs) were prepared by our laboratory and cultured in Medium 131 supplemented with 10% microvascular growth supplement, 50 U/ml penicillin, and 50 μg/ml streptomycin (Invitrogen, CA, USA). Cells were maintained in a humidified chamber at 37°C in 5% CO_2_.

A RayBiotech Human Angiogenesis Antibody Array 1000 and an Adhesion Molecule Array Q1 (Cat#QAH-ANG-2 and QAH-CAM-1) were purchased from RayBiotech Inc. (Norcross, GA, USA). Tissue microarrays of GC and paired normal counterpart tissues were obtained from Shanghai Outdo Biotech Company (Shanghai, China; Cat# HstmA150CS02).

### *In Vitro* Assays of the Angiogenic Function of Tagln2

Full-length cDNA encoding human *TAGLN2* was amplified by PCR and was cloned into the pLVX-IRES-NEO vector and verified by DNA sequencing (Invitrogen). Small interfering RNA (siRNA) targeting *TAGLN2* was synthesized by Sigma. The siRNA sequences that resulted inefficient *TAGLN2* knockdown were as follows: si*TAGLN2*#1: 5′-CCAACUGGUUCCCUAAGAA-3′, si*TAGLN2*#2: 5′-GGCAUUAACACCACUGACA-3′, and si*TAGLN2*#3: 5′-CCAACUGGCCUCUUCCUUU-3′. Plasmids (pLVX-*TAGLN2* or control vector) or siRNAs (siRNA targeting *TAGLN2* or a nontargeting siCtrl) were transfected for 36 h into EC cells *via* Lipofectamine 2000 or Lipofectamine™ RNAi MAX (Thermo, USA), respectively. Stably transfected clones were infected with lentiviral expression vector and established by G418 selection. The overexpression or knockdown efficiency was evaluated by western blotting. si*TAGLN2*#3 was used for the *in vitro* functional assays.

Cell proliferation was quantified using a CCK8 cell proliferation assay kit according to the manufacturer’s instructions. Cells were harvested and prepared for apoptosis analysis using Annexin V-FITC and propidium iodide (PI) in accordance with the instructions of the cellular apoptosis detection kit (BD) and evaluated by FACS. For Transwell migration, ECs (1× 10^6^ cells/ml in serum-free DMEM-F12) were added to the upper chambers, and DMEM-F12 supplemented with 10% fetal bovine serum (FBS) was added to the lower chambers. After 19 h, cells on the underside of the lower membrane were fixed, stained with crystal violet, and photographed.

To assess the tube-forming activity of ECs, 200 μl of Matrigel (BD Biosciences) was added to the wells and allowed to polymerize for at least 2 h at 37°C. ECs (2×10^4^) were resuspended in serum-free medium and added to the Matrigel, allowed to form polygonal structures for 6 h at 37°C, and imaged by light microscopy. Vascular branch crossing was counted in five nonoverlapping microscopic fields under 40× magnification for each condition. All experiments were performed in triplicate.

### RNA-Seq Analysis

Total RNA was extracted from HUVECs infected with lentiviral expression vector directing the expression of *TAGLN2* or with mock vectors (three replicates per group). mRNA libraries were sequenced on an Illumina sequencing platform, and bioinformatic analysis was performed by Genedenovo Biotechnology Co., Ltd. (Guangzhou, China). Differentially expressed genes (DEGs) were identified with two criteria: a false discovery rate (FDR) of < 0.05 and a |log_2_fold change| of > 1. To gain insight into the functional distribution of the DEGs, various bioinformatic analysis approaches, including heatmap analysis, Gene Ontology (GO) analysis, and Kyoto Encyclopedia of Genes and Genomes (KEGG) pathway enrichment analysis were used. A protein-protein interaction (PPI) network was constructed based on the STRING website (https://string-db.org/).

### Array Assays

HUVECs infected with lentiviral expression vector directing the expression of TAGLN2 or with mock vectors were harvested and analyzed for protein expression *via* the Human Antibody Array 1000 and Adhesion Molecule Array Q1, according to the manufacturer’s instructions. Blots were scanned with an InnoScan 300 Microarray Scanner and analyzed with ImageJ software (RayBiotech, USA). Proteins with blots densities exhibiting a fold increase of greater than 1.5 or a fold decrease of greater than 0.67 were defined as differentially expressed proteins.

### Identification of the Mechanism Mediating the Proangiogenic Function of Tagln2

To identify the important roles of the NRP1 and MAPK signaling pathways in the proangiogenic function of Tagln2, the expression levels of NRP1, two major MAPK signaling pathway molecules (extracellular signal-regulated kinase (ERK) and c-Jun N-terminal kinase (JNK)), and some differentially expressed proteins or markers involved in angiogenesis, migration and apoptosis were measured. The expression levels of *NRP1* and *TAGLN2* were modulated with siRNA or plasmid vector in Ealy926 cells stably expressing TAGLN2 and control cells, as appropriate. Then, the corresponding functional assays, including cell migration and tube formation assays, were conducted. EC samples (Ealy926 cell) with *TAGLN2* or *NRP1* expression modulation were harvested and prepared for western blot analysis. Blots were incubated with primary antibodies against Tagln2, VEGFR2, VWF, E-Cadherin, NrCAM, P-selectin, Caspase 3/cleaved Caspase 3, PARP1/cleaved PARP1 (ProteinTech, Cat#15508-1-AP, 67407-1-AP, 11778-1-AP, 60335-1-AP, 21608-1-AP, 60322-1-AP, 19677-1-AP and 13371-1-AP), NRP1, Phospho-ERK1/2, Total ERK1/2 (Abcam, Cat#ab25998, ab50011 and ab54230), Phospho-JNK, and Phospho-C-JUN (Cell Signaling Technology, Cat#4668 and 3270), respectively. Targeted proteins were detected with enhanced chemiluminescence (ECL, Millipore, USA).

### Tissue Microarray Analysis

GC tissue microarrays containing a total of 75 tumor tissues and their paired normal counterparts was used to assess the expression of Tagln2 in ECs. Immunohistochemical (IHC) staining was performed using EnVision + System-HRP kits. A transgelin-2-specific polyclonal antibody was used at a 1:7500 dilution, and antibody against CD34 (Ready-to-use, DAKO, Denmark, Cat#IS632) was used to specifically label ECs. Analysis/scoring of IHC data was performed by two certified pathologists from our hospital, and the scores were averaged. In order to evaluate microvessel density (MVD), all stained sections were screened at 40×, and vessels were counted in three spots for CD34. Any brown-stained cells or in small clusters and separate from other connective tissue elements were counted as a single microvessel, even in the absence of vessel lumen. Large vessels containing muscular walls were excluded. MVD was expressed as the mean number of counted microvessels per high power field (HPF). The expression of Tagln2 in ECs identified by CD34-positive staining was scored at 40× magnification as follows: one hundred ECs were counted per slice, and the percentage of positive cells (PP) was calculated. The intensity of staining (IS) was scored as 0: negative, 1: weak, 2: moderate and 3: strong. The results were scored by multiplying the PP by the IS (immunoreactive score = PP × IS). The relationships between the expression level and clinicopathological features were analyzed to evaluate the potential clinical value of Tagln2.

### Statistical Analysis

Statistical analysis was performed using SPSS Viewer 18.0 (SPSS, IL, USA) and GraphPad (version 5.0). Qualitative variables were analyzed by using the Kruskal-Wallis H test, the t-test and the Mann-Whitney U test. Two-tailed *P*-values of ≤0.05 were considered statistically significant. Clinical data were analyzed with Pearson correlation tests and one-way ANOVA.

## Results

### Tagln2 Protein Expression Was Aberrantly Increased in GC-Derived ECs

**Figure 1:**
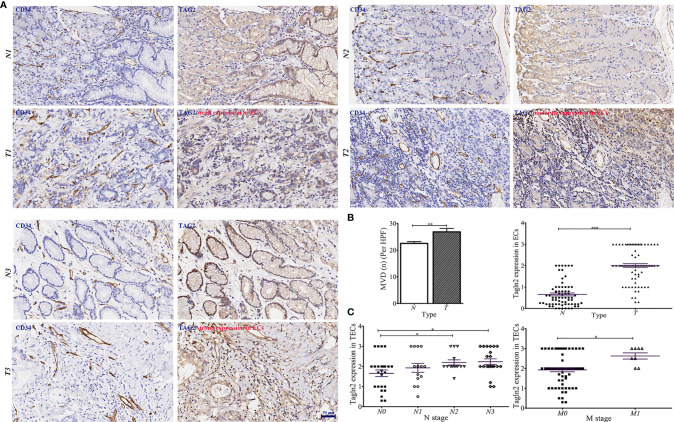
Aberrantly increased Tagln2 protein expression in tumor-derived endothelial cells (ECs) was assessed by immunohistochemical (IHC) analysis of a gastric cancer (GC) tissue microarray (TMA) chip containing 75 tumor tissues and their paired normal counterparts. **(A)** IHC images from three patients with weak, moderate and strong Tagln2 expression in ECs from GC were shown, in which CD34 was used to specifically label ECs. **(B)** The microvessel density (MVD) and IHC scores of Tagln2 expression in ECs from gastric tumor tissues (t) and paired normal counterparts (N). **(C)** The significantly positive relationship between the expression of Tagln2 in tumor-derived ECs from GC tissues and the presence of lymph node (N stage) as well as distant metastases (M stage) of patients. For the calculation of MVD, all stained sections were screened at 40×, and vessels were counted in three spots. MVD was expressed as the mean number of counted microvessels per high power field (HPF). The expression of Tagln2 in ECs identified by CD34-positive staining was scored at 40× magnification as follows: one hundred ECs were counted per slice, and the percentage of positive cells (PP) was calculated. The intensity of staining (IS) was scored as 0: negative, 1: weak, 2: moderate and 3: strong. The results were scored by multiplying the PP by the IS (immunoreactive score = PP × IS). *P < 0.05, **P < 0.01, ***P < 0.001.

GC tissue microarrays containing 75 tumor tissues and paired normal counterparts from patients with available clinical data was used for IHC analysis of CD34 and Tagln2 (**Table 1**). IHC images from three patients with weak, moderate and strong Tagln2 expression in ECs from gastric cancer were shown (**Figure 1A**). The level of CD34^+^ MVD was 26.88 ± 1.30 and 22.56 ± 0.65 in tumors and paired adjacent normal tissues, respectively (*P* = 0.004). Significant Tagln2 staining was observed in ECs from tumors (score: 2.00 ± 0.92) compared with that from normal tissues (score: 0.66 ± 0.07; *P*< 0.0001, **Figure 1B**). High Tagln2 expression (score >2) was more frequently observed in ECs from tumor tissues (68%) than in ECs from normal tissues (6%). Statistical analysis of clinical data for patients with GC revealed that sex, age, histological grade, tumor size, T stage and overall cancer stage (Stage I-IV) were not significantly correlated with the Tagln2 expression in ECs. However, the expression of Tagln2 in ECs was significantly correlated with the presence of lymph node as well as distant metastases of patients (**Figure 1C**). Tagln2 expression was positively correlated with the N stage (scores for N stage N0, N1, N2 and N3: 1.66 ± 0.17, 1.93 ± 0.22, 2.20 ± 0.13, and 2.23 ± 0.14, respectively) and M stage (scores for M0 and M1: 1.93 ± 0.10 and 2.63 ± 0.16, respectively). On the other hand, in case of the CD34^+^ MVD, no association with lymph node status or distant metastases was observed. Results demonstrated the essential function of Tagln2 in GC tumor angiogenesis, and the proangiogenic activity and mechanism involved in the process were necessary to be studied.

**Table 1:** Clinical data related to 75 gastric cancer specimens on the tissue microarray chip.

NO.	Gender	Age (years)	Histological grade	Size	T Stage	N Stage	M Stage	Stage
1	M	67	III	4.0×3.0×1.0cm	T1b	N0	M0	1A
2	M	63	II-III	2.5×2.0×3.0cm	T1b	N0	M0	1A
3	M	43	II	4.0×3.0×0.5cm	T1b	N0	M0	1A
4	M	69	II-III	5.0×4.5×1.5cm	T2	N0	M0	1B
5	M	57	I-II	3.0×3.0×1.0cm	T2	N0	M0	1B
6	F	50	II-III	5.0×5.0×1.0cm	T2	N0	M0	1B
7	M	74	II	2.0×1.2×1.0cm	T1b	N1	M0	1B
8	M	53	II-III	7.0×6.0×2.0cm	T3	N0	M0	2A
9	M	53	II	6.0×4.0×1.5cm	T3	N0	M0	2A
10	M	69	II	5.5×5.0×1.1cm	T3	N0	M0	2A
11	F	63	III	6.0×5.0×2.0cm	T3	N0	M0	2A
12	M	67	II	6.0×6.0×2.0cm	T3	N0	M0	2A
13	M	77	II	6.0×5.0×1.0cm	T3	N0	M0	2A
14	M	76	II-III	5.0×4.0cm	T3	N0	M0	2A
15	M	77	II	6.0×4.0×2.0cm	T3	N0	M0	2A
16	M	47	I-II	3.0×2.5×1.0cm	T3	N0	M0	2A
17	M	69	II	8.0×6.0×1.0cm	T3	N0	M0	2A
18	M	75	II	3.0×4.0×1.6cm	T2	N1	M0	2A
19	–	–	II	5.0×4.0×0.6cm	T2	N1	M0	2A
20	F	47	II-III	3.0×3.0×1.0cm	T2	N1	M0	2A
21	M	68	II	3.0×3.0×0.8cm	T3	N0	M0	2A
22	M	62	III	3.0×3.0×1.0cm	T3	N0	M0	2A
23	F	72	III	5.0×3.5×1.5cm	T3	N0	M0	2A
24	F	63	III	8.0×6.5×2.0cm	T4a	N0	M0	2B
25	F	73	III	4.0×3.0×1.0cm	T4a	N0	M0	2B
26	M	51	III	2.5×3.0×1.5cm	T4a	N0	M0	2B
27	F	78	II	2.0×2.0×1.0cm	T4a	N0	M0	2B
28	M	61	II-III	4.0×4.0×2.0cm	T4a	N0	M0	2B
29	M	75	III	4.0×3.0×0.6cm	T3	N1	M0	2B
30	M	55	II	4.0×2.0×1.0cm	T3	N1	M0	2B
31	M	60	II	3.6×3.5cm	T3	N1	M0	2B
32	M	61	II	6.0×5.0×0.5cm	T3	N1	M0	2B
33	M	55	II-III	2.0×2.0×0.8cm	T3	N1	M0	2B
34	M	78	III	5.0×4.0×2.0cm	T3	N1	M0	2B
35	F	50	II	2.0×1.0×0.5cm	T2	N2	M0	2B
36	M	65	III	2.5×1.5×1.0cm	T3	N1	M0	2B
37	M	80	II-III	4.2×3.5×1.5cm	T3	N1	M0	2B
38	M	68	III	5.0×4.0×1.5cm	T3	N1	M0	2B
39	M	60	II-III	5.0×4.0×1.5cm	T3	N2	M0	3A
40	F	64	II-III	4.0×2.0×1.5cm	T3	N2	M0	3A
41	M	50	II-III	4.0×4.0×2.5cm	T3	N2	M0	3A
42	F	47	III	8.0×7.0×2.0cm	T3	N2	M0	3A
43	M	55	III	5.0×5.0×1.5cm	T3	N2	M0	3A
44	M	55	II-III	4.0×3.5×2.5cm	T3	N2	M0	3A
45	M	57	II-III	13.0×6.0cm	T2	N3a	M0	3A
46	M	46	III	4.0×3.0×1.0cm	T2	N3b	M0	3A
47	M	66	III	12.0×7.0×1.5cm	T3	N2	M0	3A
48	M	55	II	5.5×4.0×2.0cm	T4a	N1	M0	3A
49	M	76	II	11.0×9.0×1.5cm	T3	N2	M0	3A
50	M	72	III	6.0×4.0×1.0cm	T3	N2	M0	3A
51	F	59	II-III	7.0×6.0×1.5cm	T4b	N0	M0	3B
52	F	64	II-III	5.0×4.0×1.3cm	T4b	N1	M0	3B
53	F	69	II-III	3.5×2.5×1.3cm	T4a	N2	M0	3B
54	M	68	II	2.0×2.0×1.5cm	T3	N3a	M0	3B
55	M	50	II-III	4.0×3.0×1.0cm	T3	N3a	M0	3B
56	M	56	III	6.0×3.5×1.5cm	T3	N3a	M0	3B
57	M	69	II-III	7.0×5.5×2.0cm	T3	N3a	M0	3B
58	M	80	II-III	8.0×7.0×4.0cm	T3	N3b	M0	3B
59	M	67	II	3.0×3.0×1.0cm	T3	N3a	M0	3B
60	M	56	III	4.0×3.0×1.0cm	T3	N3a	M0	3B
61	F	67	III	4.5×2.0×1.5cm	T3	N3a	M0	3B
62	M	72	III	8.0×6.0×1.0cm	T4a	N3a	M0	3C
63	M	65	III	4.5×3.0×1.0cm	T4b	N3b	M0	3C
64	M	51	III	8.0×7.0×1.0cm	T4b	N3b	M0	3C
65	F	65	II-III	4.0×4.0×1.5cm	T4b	N3a	M0	3C
66	M	67	II-III	6.0×4.0×2.0cm	T4a	N3a	M0	3C
67	M	30	II-III	5.0×2.5×1.0cm	T4a	N3a	M0	3C
68	M	69	II-III	5.0×4.5×2.0cm	T3	N2	M1	4
69	F	75	III	5.5×4.5×3.5cm	T3	N3a	M1	4
70	M	61	II	7.0×5.0×1.5cm	–	N2	M1	4
71	M	50	II-III	10.0×8.0×2.8cm	T3	N3a	M1	4
72	M	70	III	4.0×4.0×2.0cm	T4b	N3a	M1	4
73	M	58	II	5.5×4.0×1.0cm	T4a	N3a	M1	4
74	M	52	III	5.0×4.0×1.0cm	T3	N3a	M1	4
75	M	54	II-III	4.8×4.3×3.5cm	T4b	N2	M1	4

Gender: female (F), male (M); histological grade: high differentiation (I), moderate differentiation (II), low differentiation (III).

### Proangiogenic activity of Tagln2

**Figure 2:**
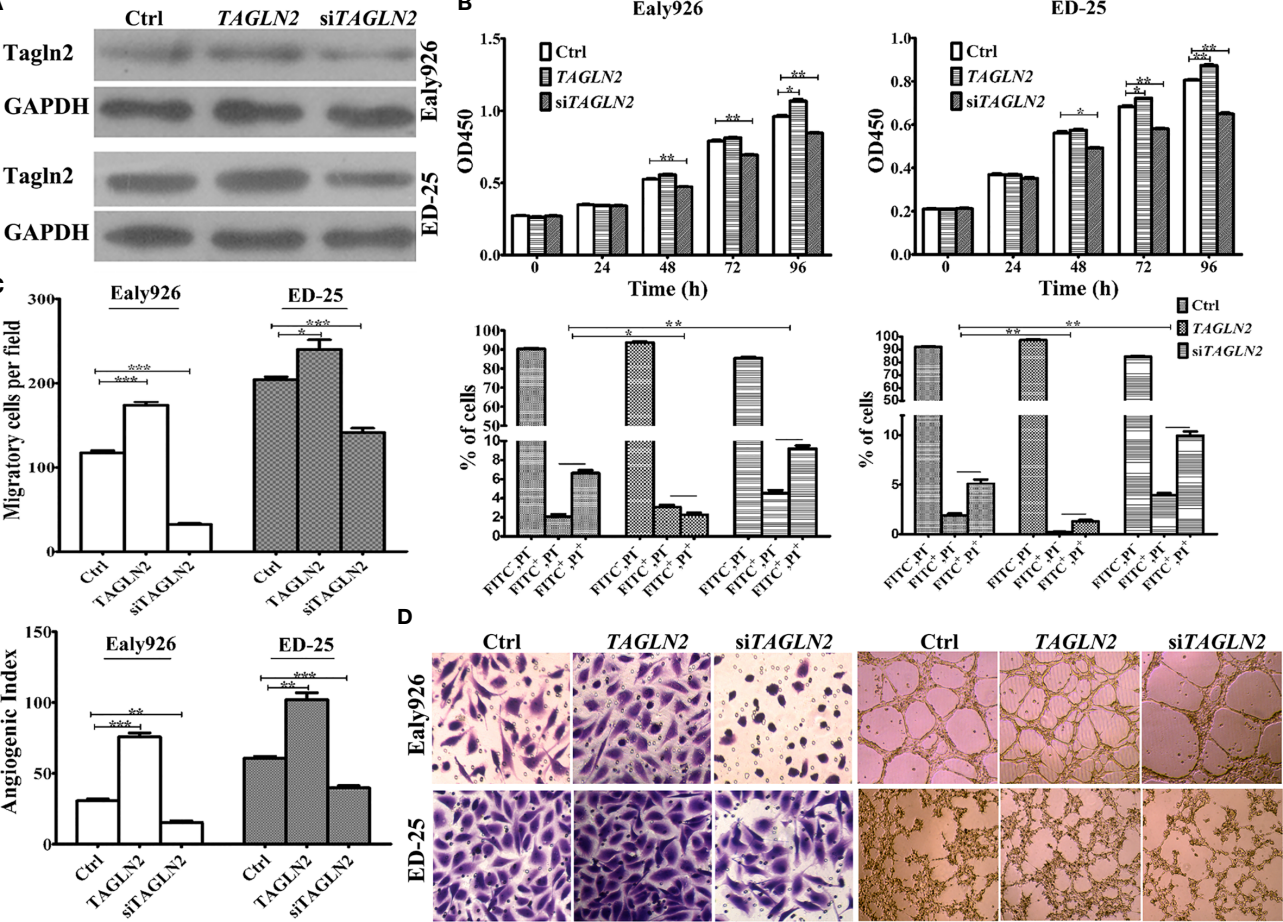
*In vitro* assays of Tagln2 functions in the Ealy926 and ED-25 cell lines with Tagln2 overexpression or knockdown. **(A)** Efficient Tagln2 overexpression or knockdown was confirmed by western blotting. **(B)** Survival and apoptosis assays with Ealy926 and ED-25 cells after Tagln2 overexpression or knockdown. **(C, D)** Migration and *in vitro* tube-forming activity of Ealy926 and ED-25 cells after Tagln2 overexpression or knockdown. Cell numbers were counted in five randomly selected fields under a microscope. *P < 0.05, **P < 0.01, ***P < 0.001.

The overexpression and knockdown efficiencies of Tagln2 in the Ealy926 and ED-25 cell lines were confirmed by western blotting (**Figure 2A**). The results of survival assays revealed that the influence of Tagln2 to the proliferation of ECs was not very significant, unless the observation time was up to 48 h. The proliferation was significantly decreased at 48 h in both cell lines after Tagln2 knockdown, but was significantly increased at 96 h in the Ealy926 cell line, or at 72 h in the ED-25 cell line after Tagln2 overexpression, respectively. Silencing endogenous Tagln2 increased the percentage of apoptotic cells (FITC^+^PI^-^ and FITC^+^PI^+^), while overexpression had the opposite effect (**Figure 2B**) at 48 h. Cell migration was significantly increased upon Tagln2 overexpression (by approximately two folds), whereas the opposite result was observed in knockdown cells. In the *in vitro* tube formation assay, with Tagln2 upregulation, the intercellular space became narrower, and many more capillary-like structures were formed; however, silencing endogenous Tagln2 resulted in abrogation of the capillary-like structures and loosening of ECs (**Figures 2C, D**).

### Tagln2-Associated Molecular Mechanism

**Figure 3:**
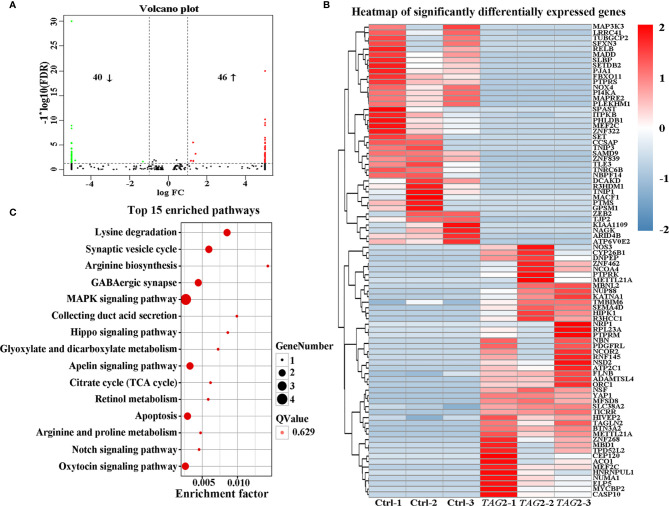
Identification the potential mechanism involved in the proangiogenic activity of Tagln2 by RNA-Seq analyses. Primary HUVECs infected with lentiviral expression vector directing the expression of TAGLN2 or with mock vectors were prepared. mRNA libraries were sequenced on an Illumina sequencing platform, and bioinformatic analysis was performed by Genedenovo Biotechnology Co., Ltd. (Guangzhou, China). DEGs were identified with two criteria: a false discovery rate (FDR) of < 0.05 and a |log_2_fold change| of > 1. A volcano plot **(A)**, heatmap **(B)** and top15 KEGG pathways enriched in the significant DEGs identified by RNA-Seq **(C)** were shown.

To obtain a comprehensive picture of the Tagln2-associated molecular mechanism, RNA-Seq analysis was performed for transcriptomic profiling. An average of 7.28 Gb of clean data were collected from each sample. The overall Q30 percentage was greater than 90.49%, and more than 92.76% of the reads were mapped to reference genes in all groups. The gene expression levels are presented as fragments per kilobase of transcript per million mapped reads (FPKM) values, and a correlation coefficient of greater than 0.99 was observed between samples from the same group. Overall, compared to the control, 86 DEGs (40 downregulated and 46 upregulated genes, **Figure 3A** and **Table 2**) were identified in *TAGLN2* overexpression group, in which 39 DEGs were upregulated by more than 8-fold. The heatmap of RNA-Seq data shows that the expression patterns were very similar across the three samples in each group (**Figure 3B**). DEGs were further analyzed by the GO and KEGG databases for functional and pathway annotations. The GO analysis results indicated that *TAGLN2*-associated genes were most enriched in the terms methyltransferase activity, protein binding, ATPase activity, ion binding, and cytoskeletal protein binding. The top 15 KEGG pathways most significantly enriched in DEGs are displayed in **Figure 3C**; the greatest proportions of DEGs were enriched in the following pathways: MAPK signaling pathway, Lysine degradation, Synaptic vesicle cycle, and GABAergic synapse.

**Table 2:** DEGs related to *TAGLN2* overexpression in HUVECs identified using RNA-Seq analysis.

NO.	Symbol	Description	log_2_^(FC)^
1	PTMS	Parathymosin	-12.46
2	FBXO11	F-box protein 11	-12.24
3	SET	SET nuclear proto-oncogene	-12.00
4	RELB	RELB proto-oncogene, NF-kB subunit	-11.82
5	TJP2	Tight junction protein 2	-11.76
6	SFXN3	Sideroflexin 3	-11.68
7	SLBP	Stem-loop binding protein	-10.92
8	NAGK	N-acetylglucosamine kinase	-10.81
9	GPSM1	G protein signaling modulator 1	-10.66
10	TNIP1	TNFAIP3 interacting protein 1	-10.59
11	TNIP3	TNFAIP3 interacting protein 3	-10.49
12	MAP3K3	Mitogen-activated protein kinase kinasekinase 3	-10.04
13	ATP6V0E2	ATPase H+ transporting V0 subunit e2	-10.04
14	CCSAP	Centriole, cilia and spindle associated protein	-9.91
15	NOX4	NADPH oxidase 4	-9.81
16	SPAST	Spastin	-9.53
17	ZNF839	Zinc finger protein 839	-9.51
18	SAMD9	Sterile alpha motif domain containing 9	-9.39
19	NBPF14	Neuroblastoma breakpoint family member 14	-9.34
20	DCAKD	Dephospho-CoA kinase domain containing	-9.23
21	SEPT9	Septin 9	-9.22
22	PJA1	Praja ring finger ubiquitin ligase 1	-9.05
23	TUBGCP2	Tubulin gamma complex associated protein 2	-9.04
24	ZEB2	Zinc finger E-box binding homeobox 2	-8.76
25	R3HDM1	R3H domain containing 1	-8.63
26	MAPRE2	Microtubule associated protein RP/EB family member 2	-8.57
27	PTPRS	Protein tyrosine phosphatase, receptor type S	-8.44
28	SETDB2	SET domain bifurcated 2	-8.38
29	ZNF322	Zinc finger protein 322	-8.26
30	TLE3	Transducin like enhancer of split 3	-8.23
31	PHLDB1	Pleckstrin homology like domain family B member 1	-8.20
32	MADD	MAP kinase activating death domain	-8.15
33	KIAA1109	Fragile Site-Associated Protein	-7.59
34	PLEKHM1	Pleckstrin homology domain-containing family M member 1	-7.59
35	TNRC6B	Trinucleotide repeat containing 6B	-7.06
36	MACF1	Microtubule-actin crosslinking factor 1	-6.87
37	PI4KA	PREDICTED: phosphatidylinositol 4-kinase alpha isoform X3	-6.57
38	ARID4B	AT-rich interaction domain 4B	-5.02
39	LRRC41	Leucine-rich repeat-containing protein 41	-4.80
40	ITPKB	Inositol-trisphosphate 3-kinase B	-1.31
41	TMBIM6	Transmembrane BAX inhibitor motif containing 6	1.17
42	FLNB	Filamin B	1.28
43	HIVEP2	Human immunodeficiency virus type I enhancer binding protein 2	1.30
44	SLC38A2	Solute carrier family 38 member 2	1.42
45	KATNA1	Katanin catalytic subunit A1	5.66
46	TICRR	TOPBP1 interacting checkpoint and replication regulator	7.06
47	CASP10	Caspase 10	7.98
48	ADAMTSL4	ADAMTS like 4	8.18
49	CYP26B1	Cytochrome P450 family 26 subfamily B member 1	8.20
50	ATP2C1	ATPase secretory pathway Ca2+ transporting 1	8.42
51	TAGLN2	Transgelin 2	8.50
52	NSD2	Nuclear receptor binding SET domain protein 2	8.54
53	YAP1	Yes associated protein 1	8.57
54	PTPRK	Protein tyrosine phosphatase, receptor type K	8.74
55	MEF2C	Myocyte enhancer factor 2C	8.74
56	ZNF462	Zinc finger protein 462	8.87
57	NRP1	Neuropilin 1	8.98
58	ORC1	Origin recognition complex subunit 1	9.06
59	NUMA1	Nuclear mitotic apparatus protein 1	9.09
60	HIPK1	Homeodomain interacting protein kinase 1	9.18
61	MBNL2	Muscleblind like splicing regulator 2	9.28
62	NSF	N-ethylmaleimide sensitive factor, vesicle fusing ATPase	9.34
63	PTPRM	Protein tyrosine phosphatase, receptor type M	9.40
64	BTN3A2	Butyrophilin subfamily 3 member A2	9.46
65	CEP120	Centrosomal protein 120	9.54
66	NCOR2	Nuclear receptor corepressor 2	9.60
67	NOS3	Nitric oxide synthase 3	9.61
68	ZNF268	Zinc finger protein 268	9.75
69	METTL21A	Methyltransferase like 21A	9.87
70	MBD1	Methyl-CpG binding domain protein 1	10.04
71	SEMA4D	Semaphorin 4D	10.07
72	ELP5	Elongator acetyltransferase complex subunit 5	10.10
73	RNF145	Ring finger protein 145	10.16
74	NUP88	Nucleoporin 88	10.20
75	NCOA4	Nuclear receptor coactivator 4	10.21
76	R3HCC1	R3H domain and coiled-coil containing 1	10.22
77	TPD52L2	Tumor protein D54	10.31
78	HNRNPUL1	Heterogeneous nuclear ribonucleoprotein U like 1	10.34
79	LMNA	Lamin A/C	10.43
80	MFSD8	Major facilitator superfamily domain-containing protein 8	10.43
81	MYCBP2	MYC binding protein 2, E3 ubiquitin protein ligase	10.52
82	NBN	Nibrin	10.80
83	ACO1	Aconitase 1	11.11
84	DNPEP	Aspartyl aminopeptidase	11.73
85	PDGFRL	Platelet derived growth factor receptor like	12.31
86	RPL23A	Ribosomal protein L23a	12.39

### Tagln2-Derived Expression of Angiogenesis Factors and Adhesion Molecular in ECs

**Figure 4:**
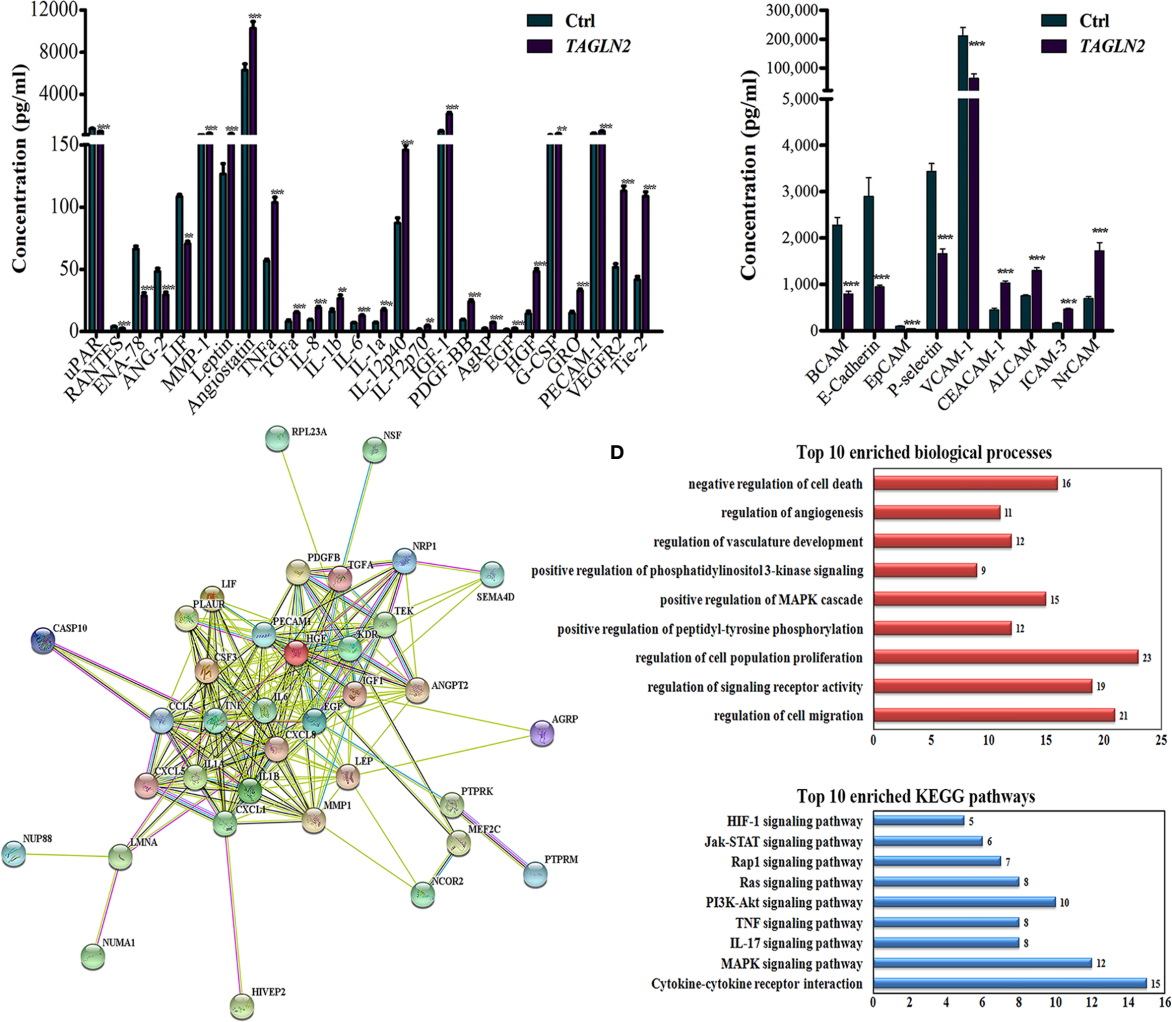
The critical molecule and signaling pathway related to the angiogenic function of Tagln2. **(A, B)** Tagln2-derived expression of angiogenesis factors and adhesion molecular in ECs. Primary HUVECs infected with lentiviral expression vector directing the expression of TAGLN2 or with mock vectors were prepared. The RayBiotech Human Angiogenesis Antibody Array 1000 and Adhesion Molecule Array Q1 (Cat#QAH-ANG-2 and QAH-CAM-1) were purchased from RayBiotech Inc. (Norcross, GA, USA). Blots were scanned with an InnoScan 300 Microarray Scanner and analyzed using ImageJ software. Proteins with band densities exhibiting a fold increase of greater than 1.5 or a fold decrease of greater than 0.67 were defined as differentially expressed proteins. The relationship between 26 differentially expressed proteins from the angiogenesis antibody array and 47 upregulated DEGs identified by RNA-Seq were analyzed and deeply mined using the STRING website (https://string-db.org/). A protein-protein interaction network was constructed **(C)**, and the corresponding Top 10 enriched biological processes and KEGG pathways **(D)** were shown. **P < 0.01, ***P < 0.001.

To detect the Tagln2-deraived changes in two main functions of ECs, namely growth factors expression and cell adhesion, angiogenesis antibody array (**Figure 4A**) and adhesion molecule array (**Figure 4B**) analyses were performed in primary HUVECs. Totally 60 angiogenesis and 17 adhesion molecular were detected, and the significantly expressed molecular were shown in **Figures 4A, B**, respectively. Five proteins involved in leukocyte recruitment (uPAR, RANTES, ENA-78, ANG-2 and LIF), were downregulated after Tagln2 overexpression. The levels of angiogenic cytokines and chemokines (TNFα, IL-8, IL-1b, IL-6, IL-1a, IL-12p40, IL-12p70, TGFα, IGF-1, PDGF-BB, EGF, HGF, G-CSF and GRO), and EC markers (PECAM1 (CD31), VEGFR2 and Tie-2) were upregulated by Tagln2 overexpression. Adhesion molecule including CEACAM-1, NrCAM (implicated in pro-angiogenic signaling, and enhance of vasculature permeability), ALCAM (acted in invasion and metastasis of several primary tumors), and ICAM-3 (identified on vessels in malignant tumors, correlated with the level of vascular differentiation) (17–19) were upregulated. However, E-cadherin (essential for morphogenetic movements), Leukocyte migration related P-selectin and vascular cell adhesion protein 1 (VCAM-1) were downregulated by Tagln2 overexpression (**Figure 4B**).

### The Most Critical Molecule and Signaling Pathway Identification

To identify the most critical molecule and signaling pathway related to the angiogenic function of Tagln2, the relationship between 46 upregulated DEGs (identified by RNA-Seq) and 26 differentially expressed angiogenesis molecules, was analyzed and deeply mined using the STRING website. A protein-protein interaction network was constructed (**Figure 4C**). The corresponding top10 enriched biological processes and KEGG pathways were also determined and are shown in **Figure 4D**. The analyzed proteins were enriched in the terms positive regulation of cell migration, signaling receptor activity, proliferation, phosphorylation, MAPK cascade, PI3K signaling, vasculature development, angiogenesis and negative regulation of cell death and were enriched mainly in the MAPK, IL-17, TNF, PI3K-Akt, Ras, Rap1, Jak-STAT and HIF-1 signaling pathways. The MAPK signaling pathway, the most important enriched pathway identified through RNA-Seq and network analysis, has been demonstrated to play crucial roles in tumor angiogenesis in previous studies (20, 21). Thirteen of the upregulated DEGs, including *HIVEP2*, *CASP10*, *PTPRK*, *MEF2C*, *NRP1*, *NUMA1*, *NSF*, *PTPRM*, *NCOR2*, *SEMA4D*, *NUP88*, *LMNA* and *RPL23A* were involved in this network. Moreover, NRP1, the node with the highest degree in the network, was involved in 9 of the top 10 biological processes and had the closest relation among the proteins encoded by the above mentioned 13 genes with proteins from the angiogenesis antibody array.

In comparison the Tagln2 associated transcriptomic profiling with that of NRP1 in our previous study (22), 20 common DEGs (23%) were obtained with the similar change trends, including 11 upregulated genes (*HIVEP2*, *SLC38A2*, *CASP10*, *ATP2C1*, *NSD2*, *PTPRK*, *ZNF462*, *SEMA4D*, *ELP5*, *NCOA4* and *DNPEP*), and 10 downregulated genes (*SFXN3, NAGK, GPSM1, TNIP1, ATP6V0E2, ZNF839, NBPF14, PJA1, TUBGCP2* and *PLEKHM1*). Moreover, four genes (*HIVEP2*, *CASP10*, *PTPRK* and *SEMA4D*) were implicated in the mentioned network. Overall, these data suggested that NRP1 may be a critical molecule and MAPK signaling pathway is likely to be the most important enriched pathway mediating the angiogenic activity of Tagln2.

### NRP1 Mediated the Angiogenic Function of Tagln2

**Figure 5:**
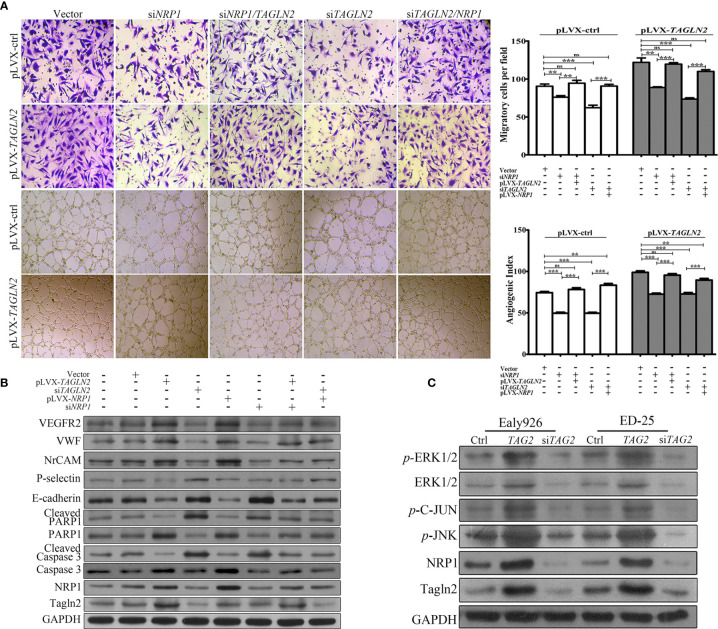
NRP1 mediated the angiogenic function of Tagln2. **(A)** Migration and *in vitro* tube-forming activity of control Ealy926 cells (pLVX-ctrl) and Ealy926 cells with stable expression of TAGLN2 (pLVX-*TAGLN2*) subjected to a series of treatments, including knockdown of endogenous NRP1, knockdown of endogenous Tagln2, knockdown of endogenous NRP1 with exogenous replenishment of Tagln2 or knockdown of endogenous Tagln2 with exogenous replenishment of NRP1. **(B, C)** After modulation of *TAGLN2* and *NRP1* expression in ECs, levels of VEGFR2, VWF (markers of endothelial cells), NrCAM (mediating capillary outgrowth), E-cadherin, P-selectin (related to ECs adhesion and migration), Caspase 3/cleaved Caspase 3 and PARP1/cleaved PARP1 (markers of apoptosis), as well as downstream targets, including phosphorylated ERK1/2, JNK, and C-Jun, were measured by western blotting. GAPDH was used as the loading control. **P < 0.01, ***P < 0.001. ns, not significant.

To examine whether NRP1 mediated the angiogenic function of TAGLN2, a control Ealy926 cell line (pLVX-ctrl) and an Ealy926 cell line stably expressing TAGLN2 (pLVX-*TAGLN2*) were subjected to a series of treatments and prepared for migration and capillary-like tube formation assays (**Figure 5A**). Knockdown of endogenous NRP1 or Tagln2 greatly decreased the number of migratory cells, and the decrease was more significant with the Tagln2 knockdown treatment. Moreover, the decrease was partially or mostly ablated by exogenous replenishment of NRP1or Tagln2 in both pLVX-ctrl and pLVX-*TAGLN2* cells. As expected, similar results were observed in the *in vitro* tube formation assay.

After modulation of *TAGLN2* and *NRP1* expression in ECs, levels of proteins involved in angiogenesis, migration and apoptosis were measured. As shown in **Figure 5B**, forced expression of Tagln2 in Ealy926 cell resulted in significant increase of NRP1, as well as VEGFR2, VWF (markers of endothelial cells) and NrCAM (mediating capillary outgrowth) protein levels by western blotting. However, corresponding levels of E-cadherin and P-selectin (related to ECs adhesion and migration), as well as cleaved Caspase 3 and cleaved PARP1 (markers of apoptosis) were decrease. As expected, inhibition of endogenous Tagln2 expression resulted in significant decrease of NRP1, VEGFR2, VWF and NrCAM, but protein levels of E-cadherin, P-selectin, cleaved Caspase 3 and cleaved PARP1 were all upregulated. These were consistent with the pro-angiogenic, pro-migration and anti-apoptotic functions of Tagln2 in ECs mentioned above. Inhibition or overexpression of NRP1 came with the consistent results, except that modulation of NRP1 expression had no impact on the protein levels of Tagln2 and P-selectin. Moreover, in NRP1 or Tagln2 silencing group, the expression of VEGFR2, VWF, and NrCAM was recovered with exogenous replenishment of Tagln2 or NRP1, and the expression of E-cadherin, cleaved Caspase and cleaved PARP1 was downregulated again, respectively. In addition, the phosphorylation levels of downstream targets, including ERK1/2, JNK, and C-Jun, were significantly increased after Tagln2 overexpression in ED-25 and Ealy926 cells, while the inverse results were found in si-*TAGLN2* cells (**Figure 5C**). Taken together, these results revealed that the molecular mechanism underlying the angiogenic activity of Tagln2—namely, Tagln2 upregulation—led to a significant increase in NRP1, which mediated the NRP1/VEGFR2 pathway and the downstream MAPK/ERK and MAPK/JNK signaling pathways.

## Discussion

The function of Tagln2 in tumor progression has not yet been clarified; importantly, the precise biological role and underlying mechanism of this molecule in tumor angiogenesis has rarely been reported. In our previous report, we found that Tagln2 was significantly overexpressed in ECs from lung cancer tumor tissues and that its high expression was associated with advanced clinical stage, increased tumor size, and histological neural invasion (16). These data indicated the important role of Tagln2 in tumor angiogenesis, so we further explored its function specifically in GC. Recently, antiangiogenic strategies have primarily focused on targeting tumor-derived ECs, which, within the tumor microenvironment, exhibit unique characteristics during tumor progression (16). The proangiogenic functions of Tagln2 were first studied in ECs. Tagln2 overexpression significantly promoted EC proliferation, motility and capillary-like tube formation, but these processes were obviously inhibited after Tagln2 downregulation. These processes are integral components of angiogenesis and prerequisites for malignant tumor growth. Tagln2 elevated the levels of angiogenic cytokines and chemokines in ECs, including TNFα, GRO, IL-8, HGF, IL-6, IL-1, PDGF-BB, and so on, which are characterized as important promoters of angiogenesis (23–26). The increased expression of the EC markers PECAM1, VEGFR2 and Tie-2 detected in the array, confirmed the Tagln2-derived angiogenesis. VEGFR2 activation is the most potent inducer of angiogenesis and is associated with enhanced EC migration and survival (27). The Tie-2 tyrosine kinase receptor, which has been shown to be the first validated tumor vascular response biomarker for VEGF inhibitor efficacy in metastatic colorectal cancer, is expressed mainly on vascular ECs and is essential for vascular maturation (28). Upregulation of the adhesion molecules CEACAM1, and ALCAM plays an important role in regulating EC network formation, migration and permeability, implicating the function of Tagln2 in the pro-angiogenesis and invasion/metastasis of tumors (17, 29). Downregulation of proteins involved in leukocyte recruitment (uPAR, RANTES, ENA-78, ANG-2 and LIF), and leukocyte trans endothelial migration (P-selectin and VCAM-1) (30), indicated the Tagln2 associated interaction decrease between leukocyte and ECs.

Furthermore, the results of integrated analysis of antibody array and RNA-Seq data suggested that NRP1 may be the critical molecule mediating the angiogenic activity of Tagln2. NRP1, a transmembrane protein, promotes both physiological and pathological angiogenesis in ECs (31). Studies in endothelial- and VEGF binding-specific NRP1 knock-in mice demonstrate that VEGF-NRP1 signaling pathways in ECs are essential for angiogenesis (32). NRP1 has been investigated mainly as a coreceptor with VEGFR2 for the VEGF_165_ isoform of VEGF-A, and its overexpression enhances the affinity labeling of VEGF_165_ (33). Then, the complex formed by NRP1 and phosphorylated VEGFR2 trigger activation of the downstream focal adhesion kinase PI3K/Akt and the MAPK pathway to promote EC proliferation and migration and angiogenic sprout formation (34). NRP1 also acts as a receptor for class III/IV semaphorins (35) and several other growth factors, such as HGF, FGF, TGF-β, and PDGFB (36, 37). NRP1 expression was upregulated in primary tumor tissue than normal tissue of stomach adenocarcinoma from TCGA data, which was related to tumor stage, and high expression of NRP1 was significantly related to a shorter overall survival (22). Recent studies have supported NRP1 as a potential tumor anti-angiogenesis therapeutic target, and NRP1-neutralizing antibodies or small-molecule antagonists have been shown to suppress tumor growth in an additive manner to VEGF neutralization (38). In the present study, the expression of NRP1 as well as that of multiple downstream signaling mediators, including phosphorylated ERK1/2, JNK and c-Jun, was significantly upregulated by Tagln2 overexpression. In addition, cells stably expressing TAGLN2 and subjected to a series of treatments, including simultaneous knockdown of TAGLN2 and NRP1, TAGLN2 knockdown with exogenous replenishment of NRP1, or NRP1 knockdown with exogenous replenishment of TAGLN2 were prepared for migration, capillary-like tube formation and protein detection assays. The results of these assays further revealed the critical role of NRP1 in the angiogenic activity of Tagln2 and the activation of MAPK/ERK and MAPK/JNK signaling pathways. These findings indicate the potential therapeutic value of Tagln2 in combating angiogenesis in GC.

Tagln2 has been demonstrated to be upregulated and possess oncogenic functions in various solid cancers (3–7), but only one study mentioned the elevated expression of Tagln2 in GC as the target of microRNA-133a (39). High expression of Tagln2 in tumor-derived ECs from lung cancer was observed in our previous research and was associated with stage, tumor size, and histological neural invasion (16). Moreover, elevated expression of Tagln2 in tumor-derived ECs but not in the whole mixed tumor tissue was reported for the first time. In this study, Tagln2 expression was significantly upregulated in ECs from gastric tumor tissues compared with that from normal tissues, and significantly correlated with the presence of lymph node as well as distant metastases. Given the substantial resources invested in NRP1-targeted anti-angiogenesis therapies for cancer, our results will provide new insights into the regulate mechanisms of NRP1 related angiogenesis and ultimately may be integral for developing new treatment strategies.

In conclusions, we demonstrated that Tagln2 expression was significantly upregulated in ECs from gastric tumor tissues compared with ECs from normal tissues and was significantly correlated with the presence of lymph node as well as distant metastases. The results of gain- and loss-of-function approaches emphasized the function of Tagln2 in promoting EC proliferation, motility, and capillary-like tube formation and in reducing apoptosis. Tagln2 upregulation led to significantly increased mRNA and protein levels of NRP1 and subsequently activated the NRP1/VEGFR2 and downstream MAPK signaling pathways to promote angiogenesis. Tagln2 may therefore be both a therapeutic target in combination with current antiangiogenic strategies targeting VEGF-mediated pathways and a candidate prognostic marker for GC.

## Data Availability Statement

The original contributions presented in the study are included in the article/supplementary material. Further inquiries can be directed to the corresponding authors.

## Author Contributions

HZ and HJ conceived and designed the research. HZ, JH, HJ, WZ, and HP developed the methodology. JH and HP analyzed/scored the IHC results. WZ and HP analyzed and interpreted the data (e.g., statistical analysis, biostatistics, and computational analysis). HZ and HJ wrote and reviewed the manuscript. JH and HP provided administrative, technical, or material support (i.e., reporting or organizing data and constructing databases). HZ and JH supervised this study. All authors contributed to the article and approved the submitted version.

## Funding

This work was supported, in part, by grants from the National Natural Scientific Foundation of China (grant number 81370048), the Natural Science Foundation of Fujian Province (grant numbers 2017J01380 and 2020CXB048).

## Conflict of Interest

The authors declare that the research was conducted in the absence of any commercial or financial relationships that could be construed as a potential conflict of interest.

## References

[1] Sung H Ferlay J Siegel RL Laversanne M Soerjomataram I Jemal A. Global Cancer Statistics 2020: GLOBOCAN Estimates of Incidence and Mortality Worldwide for 36 Cancers in 185 Countries. CA Cancer J Clin (2021) 0:1–41. 10.3322/caac.21660 33538338

[2] Chen WQ Zheng RS Baade PD Zhang SW Zeng HM Bray F. Cancer Statistics in China 2015. CA Cancer J Clin (2016) 66(2):115–32. 10.3322/caac.21338 26808342

[3] Zhang Y Ye Y Shen D Jiang K Zhang H Sun W. Identification of Trasgelin-2 as Biomarker of Colorectal Cancer by Laser Capture Microdissection and Quantitative Proteome Analysis. Cancer Sci (2010) 101(2):523–9. 10.1111/j.1349-7006.2009.01424.x PMC11159707 19930159

[4] Yoshino H Chiyomaru T Enokida H Kawakami K Tatarano S Nishiyama K. The Tumor-Suppressive Function of miR-1 and miR-133a Targeting TAGLN2 in Bladder Cancer. Br J Cancer (2011) 104(5):808–18. 10.1038/bjc.2011.23 PMC3048214 21304530

[5] Fukushima C Murakami A Yoshitomi K Sueoka K Nawata S Nakamura K. Comparative Proteomic Profiling in Squamous Cell Carcinoma of the Uterine Cervix. Proteomics Clin Appl (2011) 5(3-4):133–40. 10.1002/prca.201000077 21365771

[6] Du YY Zhao LM Chen L Sang MX Li J Ma M. The Tumor-Suppressive Function of miR-1 by Targeting LASP1 and TAGLN2 in Esophageal Squamous Cell Carcinoma. J Gastroenterol Hepatol (2016) 31(2):384–93. 10.1111/jgh.13180 26414725

[7] Han MZ Xu R Xu YY Zhang X Ni SL Huang B. TAGLN2 is a Candidate Prognostic Biomarker Promoting Tumorigenesis in Human Gliomas. J Exp Clin Cancer Res (2017) 36(1):155. 10.1186/s13046-017-0619-9 29110682 PMC5674233

[8] Zhang H Jiang M Liu Q Han Z Zhao Y Ji S. miR-145-5p Inhibits the Proliferation and Migration of Bladder Cancer Cells by Targeting TAGLN2. Oncol Lett (2018) 16(5):6355–60. 10.3892/ol.2018.9436 PMC6202496 30405771

[9] Zhao F Zhou LH Ge YZ Ping WW Wu X Xu ZL. MicroRNA-133b Suppresses Bladder Cancer Malignancy by Targeting TAGLN2-Mediated Cell Cycle. J Cell Physiol (2019) 234(4):4910–23. 10.1002/jcp.27288 30317571

[10] Xu SG Yan PJ Shao ZM. Differential Proteomic Analysis of a Highly Metastatic Variant of Human Breast Cancer Cell Using Two-Dimensional Differential Gel Electrophoresis. J Cancer Res Clin Oncol (2010) 136:1545–56. 10.1007/s00432-010-0812-0 PMC11828232 20155427

[11] Elsner M Rauser S Maier S Schone C Balluff B Meding S. MALDI Imaging Mass Spectrometry Reveals COX7A2, TAGLN2 and S100-A10 as Novel Prognostic Marker in Barret’s Adenocarcinoma. J Proteomics (2012) 75(15):4693–704. 10.1016/j.jprot.2012.02.012 22365974

[12] Leung WKC Ching AKK Chan AWH Poon TCW Mian H Wong AST. A Novel Interplay Between Oncogenic PFTK1 Protein Kinase and Tumor Suppressor TAGLN2 in the Control of Liver Cancer Cell Motility. Oncogene (2011) 30(44):4464–75. 10.1038/onc.2011.161 21577206

[13] Na BR Jun CD. TAGLN2-Mediated Actin Stabilization at the Immunological Synapse: Implication for Cytotoxic T Cell Control of Target Cells. BMB Rep (2015) 48(7):369–70. 10.5483/bmbrep.2015.48.7.132 PMC4577284 26129675

[14] Jeon BN Kim HR Chung YS Na BR Park H Hong C. Actin Stabilizer TAGLN2 Potentiates Adoptive T Cell Therapy by Boosting the Inside-Out Costimulation Via Lymphocyte Function-Associated Antigen-1. Oncoimmunology (2018) 7(12):e1500674. 10.1080/2162402X.2018.1500674 30524895 PMC6279342

[15] Kim IG Lee JH Kim SY Hwang HM Kim TR Cho EW. Hypoxia-Inducible Transgelin 2 Selects Epithelial-to-Mesenchymal Transition and γ-Radiation-Resistant Subtypes by Focal Adhesion Kinase-Associated Insulin-Like Growth Factor 1 Receptor Activation in Non-Small-Cell Lung Cancer Cells. Cancer Sci (2018) 109(11):3519–31. 10.1111/cas.13791 PMC6215889 30191639

[16] Jin H Cheng X Pei Y Fu J Lyu Z Peng H. Identification and Verification of Transgelin-2 as a Potential Biomarker of Tumor-Derived Lung-Cancer Endothelial Cells by Comparative Proteomics. J Proteomics (2016) 136:77–88. 10.1016/j.jprot.2015.12.012 26721444

[17] Nouvion AL Oubaha M Leblanc S Davis EC Jastrow H Kammerer R. CEACAM1: A Key Regulator of Vascular Permeability. J Cell Sci (2010) 123(Pt 24):4221–30. 10.1242/jcs.073635 21081647

[18] Münsterberg J Loreth D Brylka L Werner S Karbanová J Gandrass M. ALCAM Contributes to Brain Metastasis Formation in Non-Small-Cell Lung Cancer Through Interaction With the Vascular Endothelium. Neuro Oncol (2020) 22(7):955–66. 10.1093/neuonc/noaa028 PMC7339886 32064501

[19] Appleby SL Cockshell MP Pippal JB Thompson EJ Barrett JM Tooley K. Characterization of a Distinct Population of Circulating Human Non-Adherent Endothelial Forming Cells and Their Recruitment Via Intercellular Adhesion Molecule-3. PloS One (2012) 7(11):e46996. 10.1371/journal.pone.0046996 23144795 PMC3492591

[20] Tang X Zhang Q Shi S Yen Y Li X Zhang Y. Bisphosphonates Suppress Insulin-Like Growth Factor 1-Induced Angiogenesis Via the HIF-1alpha/VEGF Signaling Pathways in Human Breast Cancer Cells. Int J Cancer (2010) 126:90–103. 10.1002/ijc.24710 19569175 PMC2784023

[21] Zhuo H Zhao Y Cheng X Xu M Wang L Lin L. Tumor Endothelial Cell-Derived Cadherin-2 Promotes Angiogenesis and Has Prognostic Significance for Lung Adenocarcinoma. Mol Cancer (2019) 18(1):34. 10.1186/s12943-019-0987-1 30832661 PMC6399986

[22] Lyu Z Jin HW Yan ZJ Hu KY Jiang HW Peng HF. Effects of NRP1 on Angiogenesis and Vascular Maturity in Endothelial Cells Are Dependent on the Expression of SEMA4D. Int Mol Med (2020) 46(4):1321–34. 10.3892/ijmm.2020.4692 PMC7447310 32945351

[23] Pappa CA Tsirakis G Kanellou P Kaparou M Stratinaki M Xekalou A. Monitoring Serum Levels ELR^+^CXC Chemokines and the Relationship Between Microvessel Density and Angiogenic Growth Factors in Multiple Myeloma. Cytokine (2011) 56(3):616–20. 10.1016/j.cyto.2011.08.034 21940178

[24] Huang Q Duan L Qian X Fan J Lv Z Zhang X. Il-17 Promotes Angiogenic Factors IL-6, Il-8, and Vegf Production Via Stat1 in Lung Adenocarcinoma. Sci Rep (2016) 6:36551. 10.1038/srep36551 27819281 PMC5098156

[25] Mantovani A Barajon I Garlanda C. IL-1 and IL-1 Regulatory Pathways in Cancer Progression and Theapy. Immunol Rev (2018) 281(1):57–61. 10.1111/imr.12614 29247996 PMC5922413

[26] Xue Y Lim S Yang Y Wang Z Jensen LD Hedlund EM. Pdgf-BB Modulates Hematopoiesis and Tumor Angiogenesis by Inducing Erythropoietin Production in Stromal Cells. Nat Med (2011) 18(1):100–10. 10.1038/nm.2575 22138754

[27] Njah K Chakraborty S Qiu B Arumugam S Raju A Pobbati AV. A Role of Agrin in Maintaining the Stability of Vascular Endothelial Growth Factor Receptor-2 During Tumor Angiogenesis. Cell Rep (2019) 28(4):949–65. 10.1016/j.celrep.2019.06.036 31340156

[28] Jayson GC Zhou C Backen A horsley L Marti-Marti K Shaw D. Plasma Tie2 Is a Tumor Vascular Response Biomarker for VEGF Inhibitors in Metastatic Colorectal Cancer. Nat Commun (2018) 9(1):4672. 10.1038/s41467-018-07174-1 30405103 PMC6220185

[29] Poissonnier L Villain G Soncin F Mattot V. miR126-5p Repression of ALCAM and SetD5 in Endothelial Cells Regulates Leucocyte Adhesion and Transmigration. Cardiovasc Res (2014) 102(3):436–47. 10.1093/cvr/cvu040 24562769

[30] Goswami D Vestweber D. How Leukocytes Trigger Opening and Sealing of Gaps in the Endothelial Barrier. F1000Res (2016) 2016:5. 10.12688/f1000research.9185.1 PMC5031128 27703663

[31] Fantin A Lampropoulou A Gestri G Raimondi C Senatore V Zachary I. NRP1 Regulates CDC42 Activation to Promote Filopodia Formation in Endothelial Tip Cells. Cell Rep (2015) 11(10):1577–90. 10.1016/j.celrep.2015.05.018 PMC4528263 26051942

[32] Gu C Rodriguez ER Reimert DV Shu T Fritzsch B Richards LJ. Neuropilin-1 Conveys Semaphorinand VEGF Signaling During Neural and Cardiovascular Development. Dev Cell (2003) 5(1):45–57. 10.1016/s1534-5807(03)00169-2 12852851 PMC3918747

[33] Fantin A Vieira JM Plein A Denti L Fruttiger M Pollard JW. NRP1 Acts Cell Autonomously in Endothelium to Promote Tip Cell Function During Sprouting Angiogenesis. Blood (2013) 121(12):2352–62. 10.1182/blood-2012-05-424713 PMC3606070 23315162

[34] Nienhuser H Schmidt T. Angiogenesis and Anti-Angiogenic Therapy in Gastric Cancer. Int. J Mol Sci (2018) 19(1):43. 10.3390/ijms19010043 PMC5795993 29295534

[35] Janssen BJ Mailinauskas T Weir GA Cader MZ Siebold C Jones EY. Neuropilins Lock Secreted Semaphorins Onto Plexins in a Ternary Signaling Complex. Nat Struct Mol Biol (2012) 19(12):1293–9. 10.1038/nsmb.2416 PMC3590443 23104057

[36] Dhar K Dhar G Majumder M Haque I Mehta S Van Veldhuizen PJ. Tumor Cell-Derived PDGF-B Potentiates Mouse Mesenchymal Stem Cells-Pericytes Transition and Recruitment Through an Interaction With NRP-1. Mol Cancer (2010) 9:209. 10.1186/1476-4598-9-209 20687910 PMC2922194

[37] Glinka Y Stoilova S Mohammed N Prud’homme GJ. Neuropilin-1 Exerts Co-Receptor Function for TGF-Beta-1 on the Membrane of Cancer Cells and Enhances Responses to Both Latent and Active TGF-Beta. Carcinogenesis (2011) 32(4):613–21. 10.1093/carcin/bgq281 21186301

[38] Powell J Mota F Steadman D Soudy C Miyauchi JT Crosby S. Small Molecule Neuropilin-1 Antagonists Combine Antiangiogenic and Antitumor Activity With Immune Modulation Through Reduction of Transforming Growth Factor Beta (Tgfβ) Production in Regulatory T-Cells. J Med Chem (2018) 61(9):4135–54. 10.1021/acs.jmedchem.8b00210 PMC5957473 29648813

[39] Xu XC Zhang YH Zhang WB Li T Gao H Wang YH. MicroRNA-133 a Functions as Tumor Suppressor in Gastric Cancer. J Biol Regul Homeost Agents (2014) 28(4):615–24. 10.1007/s13277-015-3749-8 25620172

